# Understanding the Experiences of LGBTQ+ Identifying Medical Students in the UK: A Multi-Institutional Qualitative Study

**DOI:** 10.1007/s40670-026-02715-6

**Published:** 2026-04-13

**Authors:** Helen Bintley, Lisa Dikomitis, Trude Sundberg, Jolie Keemink

**Affiliations:** 1 Kent and Medway Medical School, (affiliated with University of Kent and Canterbury Christ Church University) Pears Building, University of Kent, Parkwood Road, Canterbury, CT2 7FS UK; 2 Warwick Medical School, University of Warwick, Warwick, UK; 3 School of Social Sciences, University of Kent, Canterbury, UK; 4 Centre for Health Services Studies, University of Kent, Canterbury, UK

**Keywords:** LGBTQ+, Medical school, Intersectionality, Epistemic violence, Minority stress.

## Abstract

**Introduction:**

Few studies exist exploring the experiences of Lesbian, Gay, Bisexual, Trans, Queer, and other personal identifications within the queer spectrum (LGBTQ+) medical students, particularly utilising first-person narratives. The few studies that exist illustrate an international landscape of discrimination beyond that experienced by non-LGBTQ+ peers. This study aimed to enhance understanding of LGBTQ+ medical students’ experiences in their own words to consider if and what change is needed.

**Methods:**

Utilising Poststructural and Socio-Material epistemologies and Free Association Narrative Inquiry methodology, the researchers gathered the experiences of 60 medical students from 19 medical schools across the UK and spectrum of LGBTQ+ identification. In line with the epistemologies adopted, the data was analysed using Feminist Poststructural Discourse Analysis and Materialising Discourse Analysis.

**Results:**

Structural inequalities, epistemic violence and minority stress were everyday experiences for participants. Emerging from the data were five dominant discourses related to these experiences: peers, placements, health and wellbeing, curriculum as an act of epistemic violence, and journeys and destinations.

**Discussion:**

In one of the biggest studies of its kind, the researchers demonstrated that LGBTQ+ medical students experience intersectional oppression manifesting through complex material relationships. Furthermore, we illustrated that medical curriculum was epistemically violent towards these students and, adding to the subject literature, demonstrated minority stress in this population with consequences for their wellbeing. We argue that multi-level educational interventions are needed internationally to better support these students including transparency about past oppressions, and a shift in medical school culture to one where intersectional complexity is core.

**Supplementary Information:**

The online version contains supplementary material available at 10.1007/s40670-026-02715-6.

## Introduction

There are few studies looking at the experiences of LGBTQ+ identifying medical students particularly from global majority perspectives, from students in the United Kingdom (UK) where our study was based, and utilising first person narratives [1, 2]. This, despite calls from UK Government and Stonewall (amongst many others) for improvements in services for LGBTQ+ people [3, 4] and calls for more LGBTQ+ student voices in research internationally [1]. Furthermore, recent identity-related legal challenges [5] to international Equity, Diversity and Inclusion (EDI) initiatives [6, 7] have created significant uncertainty about LGBTQ+ rights [6–8].

What is known from the data globally, which is predominantly quantitative or mixed method, is that LGBTQ+ clinicians and medical students experience significant levels of depression [9–11] bullying, self-harm, burn out [12–17] and minority stress [18] compared to their non-LGBTQ+ peers. Evidence suggests that sexual and gender minority status negatively affects promotion and career opportunities [19, 20] and the perceived need to be closeted (not openly LGBTQ+) in clinical environments. This creates the opportunity for misrepresentation of the needs of this population in medical practice, curriculum and support structures.

Our research team therefore aimed to give space to medical students who self-identify as LGBTQ + to explore their medical school experiences and understand why these experiences occur. From this, we aimed to consider how things may need to change in medical education and research and how this could happen. To support this interpretative exploration, in this and the next section we will show how Poststructuralism and Materialism formed the interpretative basis of the study and how Free Association Narrative Inquiry (FANI) methodology and discourse analyses supported interpretation of the data. Furthermore, we discuss how Queer and Feminist theoretical perspectives were utilised for context and perspective.

Other models and theories that influenced this study included minority stress, intersectionality and epistemic violence. Minority stress is a model designed by Ilan Meyer [21, 22] and is defined as ‘stress as derived from minority status’ [21]. Minority stress is a reality for many medical students and healthcare professionals globally, with consequences including closeting, ill-health, and burnout [20, 23]. This creates an environment in which patient care is potentially affected [24] and that physicians as patients do not always feel able to access care themselves, further exacerbating the problem [20, 24]. Intersectionality [25], defined as identity being ‘shaped by many diverse and mutually influencing ways’ [26], has been shown to influence how safe individuals feels in clinical spaces [27]. This includes the LGBTQ+ community with discriminatory practices, microaggressions and mistreatment common experiences for this population [28–30].

Finally, epistemic violence is defined as a failure of ‘hearers to meet the vulnerabilities of speakers in linguistic exchanges’ and is often used in relation to academic content [31]. Epistemic violence is arguably imbued with racial, socioeconomic and colonial normalised injustices [32] and this has impacts on how individuals engage with learning in clinical contexts [33, 34]. All of these lenses (and those in the next section) were utilised, to varying degrees, in this study to provide a robust interpretive understanding of the underrepresented experiences of this population, appreciating the complex challenges they face and the possibilities that a better understanding of their experiences provides for positive change in medical education.

## Methods

### Study Setting and Participants

We recruited 60 medical students (46 online interviews, 14 narrative accounts) from 19 UK medical schools between February and July 2024. This included demographic representation across the spectrum of LGBTQ+ identification (Table 1). Participants were recruited through university gatekeepers (Deans etc.), LGBTQ+ student societies, national LGBTQ+ medical organisations (The Association of LGBTQ+ Doctors and Dentists (GLADD)) and Medical Schools Council Education Leads Advisory Group (MSC ELAG).

**Table 1:** Participants’ characteristics

Participants’ Characteristics	*N*
**Sexual orientation**	
Gay	13
Lesbian	8
Bisexual	24
Queer (sexuality)	14
**Gender identity**	
Cis woman	25
Cis man	17
Non-Binary	9
Trans	5
Queer (Gender)	6
**Location of medical school**	
North England (NW, NE)	5
Midlands	2
East of England	2
South England (SW, SE)	4
Wales	2
Scotland	3
Northern Ireland	1
**Number of participants in each location**	
North England (NW, NE)	9
Midlands	7
East of England	8
South England (SW, SE)	10
Wales	8
Scotland	15
Northern Ireland	4

### Study Design and Data Collection

In this study, peoples’ lived experiences were defined as ‘unity of our situation and our attitude to it’ [35]. Ontologically, this encompassed interactions between subjects/objects and the connections that pull these subjects/objects apart or bring them together. In essence, we saw experience as ever moving and infinitely complex and we applied this fundamental perspective to the interpretation of participants’ experiences.

We utilised two different but intersecting epistemologies as the central theoretical frameworks for the study, Poststructuralism and Materialism. Poststructuralism is a concept that challenges the unifying logic and absolute truth posited by structuralist theorists such as Descartes [1]. Poststructuralism focuses on de-centring of the subject [36] and deconstructing meaning [37]. Materialism, defined as ‘the view that the world is entirely composed of matter’ [38] explores how and what the implications are of ‘things’ (objects, bodies, places, spaces) interacting and entangling [1, 39].

Poststructuralism and Materialism come from different epistemological traditions, but both embrace the complexity of experience, are inextricably linked [40, 41], and avoid interpreting experiences too narrowly, which allows different perspectives on an issue to be explored. However, by embracing complexity these approaches require the researcher to engage with uncertainty in data, to utilise their own reflexive instincts, and are arguably more epistemologically and methodologically ‘messy’ than other approaches used on similar populations.

The impact of using this multi-framework approach for such populations is, however, manifold. An example of its impact is discourse (the ways in which normative understandings of self are formed [42]). In Poststructural theory, discourse describes how societal norms such as heterosexualism make some people more likely to be socially accepted and others rejected [43–46] and this is influenced by power and politics. This is something further developed by queer theorists and termed heteronormativity [43]. However, Material perspectives provide an alternative way of understanding discursive elements by considering how entangled material relationships between objects and people (as well as many other non-human relationships) can uncover heteronormative discourses and their roots [39, 41, 47, 48]. Combining these approaches gives multiple perspectives on normative behaviours that affect individuals in myriad social contexts, and enriches understanding of the complexities of individuals’ experiences.

Additionally, we were influenced by Queer and Feminist theoretical perspectives. Feminism will be discussed as a contextual consideration in the analysis section. Current conceptions of queer identity, that being a person that identifies outside of the heterosexual, cis-gendered societal majority, arose out of political struggles related to the role of women, AIDS, and activism [49]. It is a ‘take back word’, one in which the historically derogatory nature of the term is reclaimed. This makes the concept of queer a politically evolving one and one that has been used in several contexts to explore LGBTQ+ healthcare professionals experiences [1, 2, 50].

The predominant methodology utilised in this study was Free Association Narrative Inquiry (FANI). FANI was developed by Holloway and Jefferson [52, 53] to engage with (defended, rational) subjects holistically through free association. Free association, in a research context, asks a participant to lead the conversation from which data will emerge. The methodology complemented the study aims and has been utilised in several inter-disciplinary projects [54, 55]. The approach encourages participants to engage with the emotional motivations of their narratives and draw on conscious and unconscious associations [53].

Furthermore, Holloway and Jefferson [53] argue that many forms of data collection and analysis, such as surveys, do not necessarily provide an opportunity to convey meaning in the context that participants are working/living. Even interviews, the authors argue, assume such things as shared understanding of language between researcher and interviewee, something that is potentially dangerous in terms of the marginalisation of minoritised voices. Utilising FANI enables researchers to ‘stay closer to actual life events’ [53] than by adopting more traditional methods. This twinned with the powerful association of this methodology with free association made it the best fit for this study [52, 53, 56]. This methodology adopts a two-part approach to data collection with a first interview/narrative followed by a second follow up event for clarification/triangulation. This approach created a rapport with the participants that enabled us to more authentically understand their experiences [52–55, 57].

To facilitate participants to share their stories, they were provided with interview or narrative prompts (See Supplementary Material 1). After this, written narratives (14 participants) were undertaken by participants in their own time. Online interviews (46 participants) were undertaken on Microsoft Teams at a time convenient to the participant, were video/audio recorded, and took an average of 50 minutes. We obtained informed consent from all participants. There were no incentives for participants and ethics permissions were gained institutionally and from the MSC ELAG.

For this study, we undertook extensive reflexive practice throughout data collection and analysis, which included a reflective exercise at the end of every interview (Table 2). This allowed us to reflect on first interviews honestly and appropriately prepare for follow-up events.

**Table 2:** Reflexive narrative prompts for researchers

Reflexive Narrative	Question to consider	Personal Response
Q1	What positionality did I take in this interview/reading of the narrative?	
Q2	What associations did I make with this participant's experiences?	
Q3	What challenges did I face with this participant?	
Q4	Is there any further work to be done? (Discussion, referral etc.)	

### Data Analysis

We undertook successive rounds of immersive Feminist Poststructural Discourse Analysis (FPDA) [58, 59] and Materialising Discourse Analysis (MDA) [60]. This included a denotative (‘factual’ description) and connotative (interpretive analysis) analysis in line with Baxter’s [58] FPDA. For the denotative analysis we wrote a concrete account of the events in the interviews/written narratives. For the connotative analysis our interpretation predominated Poststructural deconstructionism, polyphony (how voices interact) and hyperglossia (making evident the silenced voices). This involved searching for social discourses emerging from the data, metalanguage (how participants describe their speech), intertextuality, and the associated ‘matrices of communicable utterances’ [58].

For MDA, analysis included Material consideration of the politicisation of objects and adopted a new materialist perspective in line with associated studies [41, 60]. For both analyses a set template was used (Tables 3 and 4), which supported researchers to undertake separate analyses of the data using both approaches, after which the two analyses were compared to identify the final discursive outcomes. This involved multiple rounds of immersive analysis, which enabled discourses to emerge through subject repetition and impact on participants.

**Table 3:** FPDA analytic considerations

**Denotative Analytic Approach:**
Concrete account of the events occurring in the speech event. Verbal and non-verbal interactions. Awareness that still a cultural construction through analysis.
**Connotative Analytic Approach:**
Searching, interpretive, institutional and social discourses existing in the research area. Metalanguage – how participants describe their speech. Be aware of intertextuality – foregrounding and highlighting discourses that are influenced by other discourses and how.
**Specific Considerations during analysis:**
Deconstructionist: textual interplay, fact replaced by representation, no fixed meaning, process creates structure, avoid voice privilege, critical of assumptions, connections between opposites.
Polyphony: multiple voices analysed together.
Hyperglossia: making evident the silenced voices.
Synchronic: micro-analysis of short discussions/ discussion moments – particularly in relation to power shifts.
Diachronic: the language of individuals, groups, communities over time.
**General Considerations during analysis:**
What emerges/ words and speech events/ themes and preoccupations/ links/ our relationship in convo with the following in mind – emancipation/ liberation/ oppression/ silencing/ control/ experience as complex and socially constructed/ power and how it shifts/ decentring subject/ dominant/ non-normative discourses/ privilege/ refs to gender, sex and sexuality
Speeches that illustrate intersections/ tensions/ opposites/ contradictions/complements/ of different discourses especially in relation to FP lens – emancipation/ liberation/ oppression/ silencing/ control/ experience as complex and socially constructed/ power and how it shifts/ decentring subject/ dominant/ non-normative discourses/ privilege/ refs to gender, sex and sexuality

**Table 4:** MDA analytic considerations

**Analytic approach:**
(In line with Van Eeden P. Materialising discourse analysis with James, Schmitt and Latour. Humanities and Social Sciences Communications. 2017;3:17039)
Turn towards materiality in the emerging data.
Turn from a post-structural appreciation of the data but do not turn away from the influence of discourse on material understanding.
Consider the politicisation of participants, actants, emerging data sources
Turn towards and consider a new materialist perspective (turn to matter, power of matter, relational entities, turn away from representation, essentialism, and anthropocentrism).
**Specific Considerations during analysis:**
Inter-objectivity/ sticky/ entangled relations of things/ tentacularity
Posthuman performativity, agential realism, intra-actions
Constantly shifting networks of relationships/ nothing exists outside of those relationships
Entanglements as dialectic relationships (dependence and dependency), Tanglegram and formal networks
**General Considerations during analysis:**
What emerges/ words and speech events/ themes and preoccupations/ links/ our relationship in convo with the following in mind - Posthuman performativity/ materialist decentring human/ inter-intra actions of objects/ materials/ things/ people/ non-representational things/ what things emerge from the data that illuminate our understanding of LGBTQ+ medical student experience?
Speeches that illustrate intersections/ tensions/ opposites/ contradictions/complements/ of different discourses especially in relation to materialist lens - Material decentring/ posthuman performativity/ tensions/ contradictions/ complements/ inter-intra actions/ time and matter relations/ things, people, environment/ relationality/ critically appraising the importance of the human experience/ agency, autonomy/ agential realism/ discourses emerge from intra actions as opposed to being an outcome per se.

**Diagram 1:**
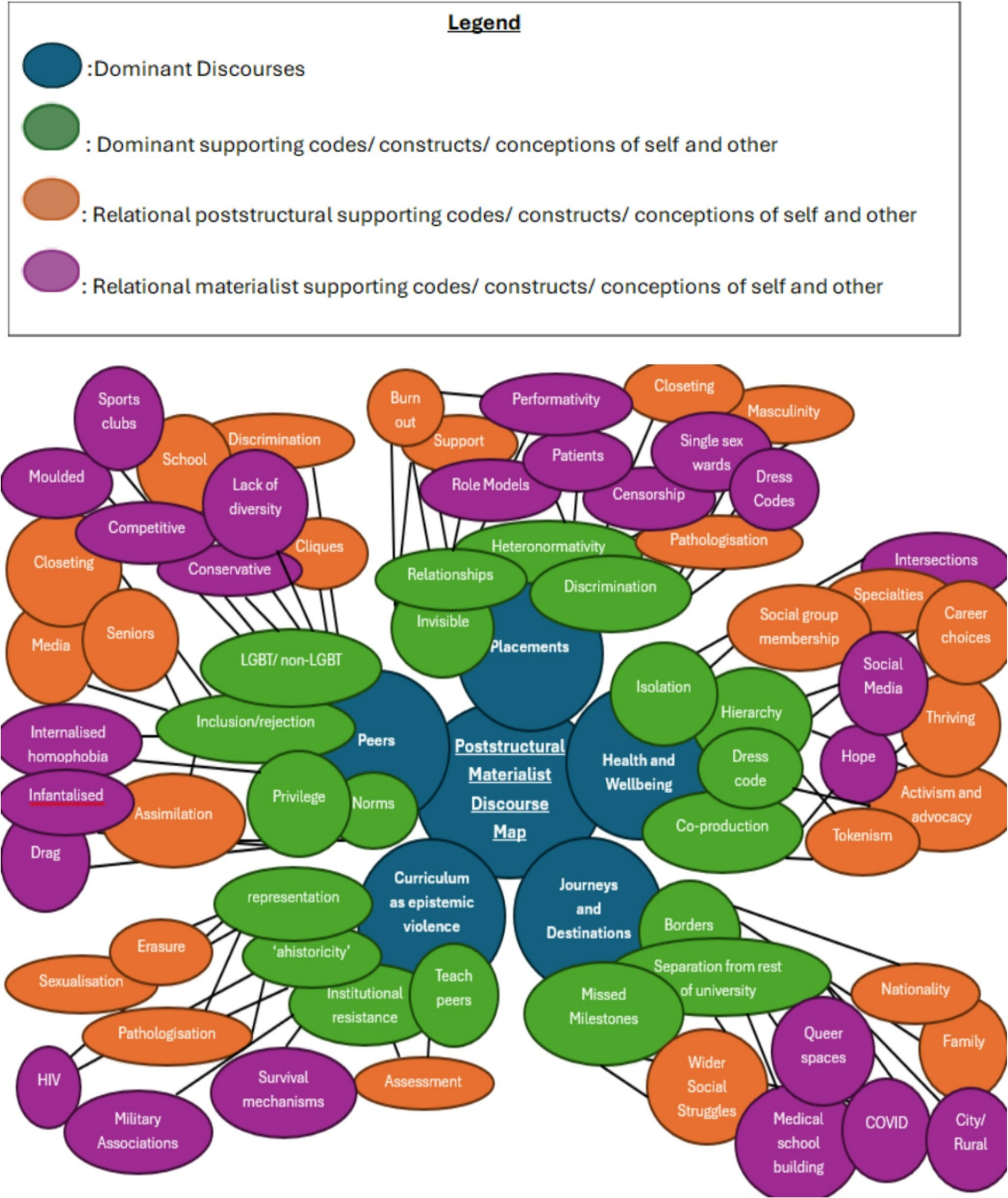
Poststructural materialist discourse map

From this emerged a discourse map, which illustrated the final overlapping Feminist Poststructural and Material discourses (this can be found in Diagram 1 in addition to a summary of the outcomes (Table 5). This map was constructed using applied examples of discourse analysis and mapping. Discourse mapping is an inter-disciplinary method of engaging with text, ideas and experience on a micro, meso and macro level [61–64]. This allows for understanding of events that are complex, unclear but influential on people’s lives [61]. This approach enables visualisation of such complexities and the codes/constructs/conceptions of self and other that contribute to associated discourses.

**Table 5:** Discourses and relational codes/constructs/conceptions of self and other

Dominant Discourse	Dominant supporting codes/constructs/conceptions of self and other	Relational Poststructural codes/constructs/conceptions of self and other	Relational Material codes/constructs/conceptions of self and other
Peers	LGBTQ+ versus non-LGBTQ+ peers Inclusion or rejection by social groups Privilege of some peers over others Normative behaviours at medical school	Media representation of LGBTQ+ people Impact of school before medical school Closeting as a consequence of discrimination Assimilation in order to fit in Perceptions of seniors as non-inclusive Cliques and biomedical mindsets Understanding difference as other Discrimination	Cookie cutter/moulded students and doctors Lack of diversity amongst peers Competitive and conservative Infantilization of medical students Internalised bi/homophobia Sports clubs as sources of inclusion and othering LGBTQ+ peers flock together Drag culture
Placements	Invisible or unfathomable by others Relationships with others Heteronormativity Discrimination	Queer friendly/unfriendly specialties Hierarchy of pathologisation Student Support Closeting Performativity Burn out Power, privilege, masculinity Compulsory heterosexuality	Self-censorship Single sex wards as non-inclusive Impact of LGBTQ+ identity on patients Role models Dress code as violent Adaptation of performativity to specialty Irrelevance, invisibly queer
Health and Wellbeing	Isolation of LGBTQ+ individuals Hierarchy at medical school Dress code as a violent act (dominant supporting codes/construct here) Co-production between staff and students	Career choices based on identity Sadness, fear, isolation, hate Thriving despite challenges Hope LGBTQ+ activism and advocacy ‘Fringe’ members of multiple social groups Students as drivers of social change in medical school Tokenistic curriculum developments	Social media and sense of self Intersection of LGBTQ+ identity and being a medical student Material objects – lanyards, pronoun badges, scrubs Communities of support and intersectionality
Curriculum as an Act of Epistemic Violence	Representation in curriculum and medical school Ahistoric nature of curriculum Institutional resistance to change Expectation that LGBTQ+ students will teach peers about their identities	Scared/fear of curriculum as something that is harmful to LGBTQ+ people Lack of LGBTQ+ content and erasure of LGBTQ+ people from curriculum Lack of discussion about EDI Assessment and heteronormativity Ahistoric curriculum Sexualisation and pathologisation of LGBTQ+ identity	Wellbeing services not always catered to LGBTQ+ students Survival mechanisms needed when engaging with curriculum Militaristic associations Intergenerational HIV trauma
Journeys and Destinations	Borders Separation from the rest of the university Missed social milestones for LGBTQ+ people	International perspectives on students’ challenges Intersections between race, politics, culture and identity Understanding of wider societal struggles Family	Movement across borders Culture and cultural change across borders Medical School buildings City versus rural settings Queer spaces not being owned by LGBTQ+ medical students COVID

Other analytic approaches were considered for this study including Foucauldian Discourse Analysis and Critical Discourse Analysis. However, these options neither embraced the schismatic, ‘productive friction’ [58] of FPDA nor included an overt appreciation of the material discursive perspective. Therefore, FPDA and MDA were deemed the best options for considering this multifactorial issue from multiple perspectives.

Feminist theory was also utilised during data analysis, which is traditionally considered to fall into three ‘waves’ and draws on diverse lenses. Mills [65] identifies a number of features of the most recent third wave of feminism, which include the performative nature of gender, power, and resistance. This sense of resistance and a move towards change [58] aligns with the aims of this project and the researchers’ positionalities relating to social justice. Related to this, feminist poststructuralism was a vital component of the analytic process. Feminism and poststructuralism are not natural partners epistemologically. However, Baxter [58] argues that uncritical use of terms such as sex and gender in feminism is fundamentally un-feminist and Poststructuralism provides a way that intersectional feminist oppression can be explored critically. The contradictions, discomfort, and ‘friction’ of bringing together these concepts enables a new conversation about difficulty. Feminist Poststructuralism then, is a space of ‘schism’ where new knowledge is possible.

Reflexively, going into this study, we understood that our positionality had an impact on data collection and analytic choices. This study is conceptually based around the need to understand the experiences of these individuals in order to move towards something better that aligns with a social justice agenda. With this comes a unified understanding amongst the research team that a problem exists (LGBTQ+ medical students and doctors experience inequality), that this is a social justice issue and that this needs challenging. This aim comes from extensive experience of working with the LGBTQ+ community in a range of healthcare education settings as well as having experience of using the methods described in a range of contexts.

## Results

This study aimed to enhance understanding of LGBTQ+ medical students’ experiences, with UK medical students as its focus. Five dominant discourses emerged from the analysis which are illustrated in Diagram 1, Table 5 and detailed below.

### Peers

Peers were identified as a dominant discourse with supporting codes/constructs/conceptions of self and other including differentiation between LGBTQ + and non-LGBTQ+ peers, privilege, normative expectations, and community inclusion and rejection. All of these supporting codes/constructs were identified by the majority of participants across the spectrum of LGBTQ+ identification, medical school location, and year of study.

Peers were described by many participants as either LGBTQ + or not, and as inaccessible and conservative if not. Non-LGBTQ+ peers were seen by some as privileged and Materially “moulded” (Student East England) to become a doctor that looked (normatively gendered), acted (adopting a medical gaze), and sounded a certain way (British Received Pronunciation). These elements were perceived to contribute to minority stress, othering of LGBTQ+ medical students, internalised homo-bi-transphobia and rejection of their LGBTQ+ identity in order to “fit in” (Student East England). A participant described:

“It’s always that sort of like heteronormative assumption that yeah, you’re straight sort of thing. It felt like almost like I’m going back into the closet almost” (Student East England).

Participants described overt discrimination by non-LGBTQ+ peers but by contrast LGBTQ+ peers were a community, which fostered a “sense of belonging” (Student North England). These contrasting relationships between LGBTQ + and non-LGBTQ+ peers affected participants’ health and wellbeing. This manifested through isolation for some participants because ‘peers would kind of reject me based off of my sexual orientation’ (Student South England). This contrasted with the sense of belonging within the LGBTQ+ community, which was repeatedly described as being ‘surrounded’ (Student Scotland) by people who were ‘really accepting’ (Student Midlands).

Despite this sense of belonging, participants had concerns about the LGBTQ+ community at medical school. Exclusionary behaviours were repeatedly described including ‘some LGB people can leave out the T sometimes and this is obviously unacceptable’ (Student North England). Another participant described that:

“Queer spaces can be not fully inclusive, for example not including people of colour or transgender people. There is definitely room for improvement” (Student North England).

Despite these concerns there was a perceived need for a ‘unified front’ (Student East England) in the LGBTQ+ community at medical school by many because ‘this country and others are regressing in their views’ (Student North England) and this made many participants feel at risk of discrimination.

### Placements

Placements were identified as a dominant discourse with supporting codes/constructs/conceptions of self and other including discrimination, heteronormativity, varying experiences with healthcare stakeholders (i.e. patients, healthcare professionals), invisibility and irrelevance. Many of these supporting codes/constructs were identified by the majority of participants across the spectrum of LGBTQ+ identification, medical school location, and year of study.

The majority of participants found clinical placements challenging with evidence of discriminatory practices. One participant described being, “met with a barrage of insensitive questions underlined with a lot of microaggressions” (Student Midlands). This created an environment in which heteronorms were not challenged leaving participants with “a lot of social dysphoria” (Student North England) and minority stress, and this prevented them from reaching out to the medical school about concerns.

Participants were keen not to talk about patients in a negative manner, but a persistent concern was the impact of being outed on their professional relationships with patients. Another participant explained:

“I don’t want people to ask questions because it’s a professional like environment … I don’t want like patients to guess… I just don’t want to put myself in a position where I could be hate crimed” (Student North England).

In relation to healthcare professionals on placements, there were multiple examples of inspiring as well as concerning interactions. One participant described how working with a surgeon undertaking gender affirmation surgery had been ‘so inspiring and affirming’ (Student East England). However, many participants described negative interactions with healthcare professionals. In particular, Obstetrics and Gynaecology (O + G) was repeatedly described as a heteronormative specialty. Participants described the ‘complete neglect’ (Student Wales) of the LGBTQ+ community in O + G with ‘no room made for any er anyone who … wasn’t a cis man or woman’ (Student Midlands). This manifested in awkward interactions with healthcare professionals where they repeatedly failed to ‘neutralise’ (Student Midlands) their language reinforcing heteronormativity and binarism.

Participants also repeatedly described themselves as invisible on placement. LGBTQ+ medical students described needing to be “invisibly queer” (Student Scotland) because “a patient yelling a transphobic slur at me” (Student North England) were not uncommon experiences and this affected their engagement with clinical learning. Another participant used a planetary metaphor to describe their perceived irrelevance in the clinical team, describing themselves as a “speck of dust” in relation to the “planets” (Student South England) that were clinical staff.

### Health and Wellbeing

Health and wellbeing was identified as a dominant discourse with supporting codes/constructs/conceptions of self and other including isolation, hierarchy, dress code, overt discrimination and co-production. All of these supporting codes/constructs were identified by the majority of participants and from participants across the spectrum of LGBTQ+ identification, medical school location, and year of study.

Health and wellbeing in the participant population was consistently associated with isolation and exclusion. Exclusion manifested in peer groups and social groups (cultural, religious, LGBTQ + etc), leaving them on the ‘fringes’ (Student East England) of these groups. This also manifested in the ‘very hierarchical structure’ (Student Scotland) of some specialities, with some participants aiming to remain invisible in such specialties to avoid perceived repercussions. One participant explained that:

“I have seen … surgeons being very transphobic to patients … and so it’s almost a bit of a don’t ask, don’t tell situation going on” (Student Scotland).

Despite this, some specialties (General Practice, Sexual and Reproductive Health and Psychiatry) were considered LGBTQ+ friendly and although difficult to find, role models were particularly important in this context. One participant explained that, ‘It is so important to see people who look like you in senior roles and succeeding in their careers’ (Student Wales).

Many participants highlighted an association between LGBTQ+ identity and dress codes. Dress codes in medical schools describe written expectations of professional dress for clinical teaching contexts but there is no unified inter/national agreement of dress codes [66]. One participant explained that dress code violations ‘describe a queer person!’ (Student Scotland) and other participants described the intersections of politics, and religious and cultural norms as being ‘stigmatised’ (Student North England) through dress code, which erased or actively discouraged natural hairstyles and religious dress requirements. One participant explained, ‘So I can’t wear a [Palestine] pin that shows I’m Muslim, but what about my hijab?’ (Student South England). Another participant explained that for them, from a socioeconomic perspective:

“Dress code is an important issue for a lot of people … I don’t feel my identity has expressed that greatly in how I dress … other than the stress of having to find the money for like nice clothes for placement” (Student Scotland).

One solution proposed by several participants was that medical students should wear scrubs. One participant explained that scrubs, ‘takes this dress out of like having to wear nice clothes to placement’ and are ‘the great leveller’ (Student Scotland). Another participant explained that ‘I’ve never had any issues with patients … but I suppose we all look the same don’t we, we’re all wearing scrubs’ (Student North England).

Whatever the solution, participants felt that the onus was on them to enact meaningful change. This manifested through co-production of curriculum change, that being the process of productively sharing responsibility for curriculum change between students and teachers [67]. However, in antithesis to its intention, participants described accounts of unsupported co-production. One participant explained:

“Yeah I’ve done a lot of co-production … co-production really doesn’t involve the student as an equal because oftentimes they’re [academic staff] very … ruthless … they just cut a lot of our work …” (Student South England).

Despite this, most participants felt they had ‘freedom and joy’ (Student South England) at medical school. This was driven by material relationships between hope, role models, material LGBTQ+ objects and ‘a good community’ (Student Scotland).

### Medical Curriculum as an Act of Epistemic Violence

Curriculum as an act of epistemic violence was identified as a dominant discourse with supporting codes/constructs/conceptions of self and other including representation, expectations of LGBTQ+ students to teach others about queer identity, intersectional ‘ahistoricity’, and resistance to change. All of these supporting codes/constructs were identified by the majority of participants and from participants across the spectrum of LGBTQ+ identification, medical school location, and year of study.

LGBTQ+ representation in curriculum, especially in the ‘case studies or practice patients’ (Student South England) (vignettes, Problem Based and Team Based Learning cases) lacked intersectional perspectives and was not challenged effectively. One participant described, ‘I seem to be actively not in this curriculum’ (Student Wales). Another participant was told that, ‘they don’t need to teach us about queer healthcare because it’s not that different from health care for other people’ (Student South England). This potential (through lack of knowledge about specific LGBTQ+ related patient need) and actual (erasure) violence had a significant impact on students, and resistance to change from the academy meant ‘nothing ever changed’ (Participant South England).

Materially, other participants noted that trans patients, in particular, were either ‘plopped’ (Student Wales) into teaching vignettes tokenistically or were often ‘killed off’ (Student North England) which gave the impression that trans and non-binary individuals were easily disposable. Participants felt these approaches paralysed meaningful conversation about difference and increased the likelihood of students closeting at medical school. Another participant explained:

“It was the most dramatic ending [to the case], he died in like three seconds and I was thinking, ‘Why does it need to be like that?’ (laughter) … I find myself not getting too comfortable … it reminds me of like how people can actually be” (Student North England).

Furthermore, many participants were expected to teach other students about their lives and LGBTQ+ healthcare. Another participant described how they:

“Do some lectures for the first and fourth years because ‘it’s fallen to us, which just feels like it’s putting a lot of extra work on the minority group who are being discriminated against, right?” (Student Scotland).

Underscoring these descriptions was a sense of intersectional ‘ahistoricity’ (Student South England) related to medical curriculum. Participants felt that there was a profound lack of historical context in curriculum which created a ‘hierarchy of pathologisation’ (Student Wales) where historically stigmatised groups were persistently pathologized (the action of regarding something as pathological’ [68]) compared to non-stigmatised individuals. Alternatively, other participants described the curriculum as steeped in history but one that perpetuated historical associations with medical education making the current medical curriculum seem ‘horrendously outdated’ (Student South England). This was reflected Materially, in textbooks, which were perceived to be white-cis-heteronormative and inaccessible in terms of language. One participant noted that:

“A lot of the types of textbooks you use they’re old, right? … And so, I think that the professors they maybe trained 20, 30, 40 years ago.So, there was possibly no training that happened between then and now to keep them updated on how their language affects people” (Student South England).

## Journeys and Destinations

Journeys and destinations were identified as a dominant discourse with supporting codes/constructs/conceptions of self and other including physical and representational borders, separation and isolation from the rest of the university, and missed milestones. All of these supporting codes/constructs were identified by the majority of participants and from participants across the spectrum of LGBTQ+ identification, medical school location, and year of study.

Journeys were described in relation to borders, be that physical or metaphorical, including the ‘geographical barrier of the sea’ (Student North England) and were associated with emerging understandings of participants’ intersectional identities. Participants described moving from one border to another as a movement across ‘cultural lines’ (Student East England). This was twinned with the perception of the medical school as protective, in that it provided a reason (that being the pressure to succeed) not to engage with factors that were uncomfortable. One participant explained that:

“Growing up where societal acceptance of the LGBT+ community lagged behind … has shaped much of my experience. Living away from my family … coupled with the demanding schedule of medical school has limited my ability to maintain close family ties. Medical school is my life …” (Student North England).

In terms of destinations, many participants felt that the medical school and clinical buildings were entangled with 'feeling isolated from the rest of the university' (Student North England). Participants described the ‘segregation’ (Student Wales) of the medical school building as preventing cohesion of queer communities in universities. One participant explained that:

“The medical school is a bit separate from main campus unfortunately … despite us having about 200 students per year, it, it is still a lot smaller student body and people don’t have quite as much time … to invest in their student community and … build that support network and community” (Student Midlands).

This concept of buildings being inclusive or exclusive extended to self-identified queer spaces for many participants. Queer spaces were destinations that were ‘not owned’ (Student East England) by queer students. By this the participants did not mean physical ownership but that spaces were not ‘queered’. Spaces used for queer events were often shared within an otherwise ‘straight environment’ (Student North England). This had an impact on participants’ perceptions of safety and openness. One participant explained that at their university there are:

“a few gay bars erm and by gay bars I mean one of them does a student night on a Tuesday. So, it’s not, it’s just a load of straight people” (Student Northern Ireland).

Journeys were also described in relation to the COVID-19 pandemic and ‘missed social milestones’ (Student East England) where the desire to ‘fit into a box’ (Student South England) meant their normative social milestones, inclusive of their LGBTQ+ identity, were not met. Many described the pandemic as a life-changing journey with some having had to return home and navigate the barriers that had encouraged them to leave in the first place. Others returned home with a new perspective on self and other, and this caused conflict and resolution with family and friends in equal measure. One participant explained that:

“Things were initially fine because everyone thought it was going to be short term … and then slowly descended into realising that my mum had an issue with me being with a woman…so then the rest of my COVID isolation was just pretty horrific” (Student Wales).

Others found the pandemic isolating, describing people being unable to see partners, and a move to exclusively online queer spaces. One participant noted that, ‘first year is already kind of tough but not having any peer support for it … it impeded on socialising a lot’ (Student East England).

Finally, for many there was an appreciation for the journey ongoing. The journey of understanding themselves was not static, and many participants did not see themselves as a ‘fixed’ gender or sexual orientation but one that encompassed ongoing growth. Furthermore, there was less of a concentration on the destination, a desire to be labelled, and more on a desire to be understood as a complex individual. In essence, the journey appeared to provide participants with a language with which to talk about difference.

## Discussion

This study filled a gap in the literature by developing understanding about the experiences of LGBTQ+ medical students using first person narratives with a large sample. We revealed that LGBTQ+ medical students’ lives are complex and entangled with Material, intersectional difficulty that are influenced by peers, placements and journeys. We also discovered that students found supportive communities that brought joy, hope, and helped them thrive despite these challenges.

Our findings revealed that medical curriculum appears to be epistemically violent towards LGBTQ+ people manifesting through power imbalances, heteronormativity, and erasure in multiple contexts. Finally, adding to the existing literature, we demonstrated interconnected examples of minority stress with associated consequences for health and wellbeing. We argue that these elements highlight possibilities for positive change in medical education and research internationally.

### Material Intersectional Complexity

The role of a medical student is a complex one. Medicine is arguably a vocation and involves a re-conceptualisation of self as members of communities of practice [69]. This involves a complex evolution of self-understanding in terms of personal and professional identity formation and involves socialisation, including negotiation and rejection, of existing personal identities [69]. This evolution can create dissonance in terms of individuals’ roles as students, (often) emerging adults and future physicians [70].

For the participants in our study, this dissonance created difficulty related to their emerging understanding of themselves as LGBTQ+ people in addition to these existing self-understandings. This manifested in several ways, one being their Material relationship with the medical school building. Perceived to be physically and discursively (through value/funding/prestige) separate from the rest of the university, the medical school building created uneasy tensions between LGBTQ+ medical students and the wider university. This was entangled with power, socioeconomics, privilege, and colonialisation all of which affected their sense of self in this space. Similarly, in their study on the impact of a new medical school building on student experience, Hawick et al. [71] found that medical students felt trapped by the medical school building and disconnected from the wider university. LGBTQ+ medical students then, especially those at risk of intersectional oppression, have the potential to be particularly negatively impacted by these difficulties because the aforementioned dissonance is emerging for them on intersecting plains be that personal or professional identity, sexual orientation and/or gender identity.

Despite this, it can be argued that medical school buildings are important for shaping students’ professional identities as future caregivers. As an example of this, Nott [72] examined the interplay between medical architecture, embodied learning, and historical understandings of the body in medical education. The author identified the symbiotic relationship between these elements, and that material engagement with the medical school building itself profoundly affects learners’ understanding of their professional bodily knowledge as well as that of their patients. This sense of professional identity manifests through material objects such as medical art on the walls of these buildings and simulated clinical spaces that encourage identification with the standards of the medical profession [72]. This complex relationship between student and emerging future physician, Nott [72] argues, reminds medical students of professional expectations. Despite this, it is important to acknowledge that bodily experience is connected to power, representation and in/equity, and this has the potential to impact how medical students perceive themselves and their patients.

Another example of material, intersectional oppression from this study was dress code. Dress code was defined earlier in this article and there are associations between dress code and white, masculine social norms [73], microaggressions and marginalisation in clinical education settings [74–76]. For many of our participants there were additional associations between dress code and un/professional behaviour, dirt/pathogens, racist norms, politicisation and sexualisation of identity. Expectations of medical student dress is complex and further entangled with patient expectations and clinical priorities internationally [77, 78]. Medical environments need to reduce the risk of iatrogenic infection, make patients feel safe, and medical professionals need to be identifiable [78, 79]. However, what this presentation looks like very much depends on individual patients and therefore expectations vary [77]. As examples, surgical scrubs have been shown to be acceptable professional dress by some patients [78] and unacceptable by others [80], and a randomised control trial [81] found that doctor’s attire did not affect perceptions of compassion or professionalism at all by patients receiving palliative care.

This variation in patient expectation twinned with the impact of dress codes on marginalised groups calls into question the need for flexibility in dress codes at medical school. Regularly reviewed inclusive dress codes [75], bias awareness training [76] and, as suggested by our participants, ‘listening meaningfully’ to those affected could be ways in which this could be realised. What is needed more generally, we argue, are interventions that address the issue of material complexity, and multilevel cultural changes that are specific to medical contexts. We suggest these tensions should be considered inter/nationally when medical schools are built and education policy is re-developed.

### Medical Curriculum as an act of LGBTQ+ related Epistemic Violence

This study revealed that UK medical curriculum was epistemically violent towards participants. Undergraduate medical curriculum is understandably rigorous with a necessary, difficult balance maintained between scientific education and person-centred learning [82]. Medical curriculums are intended to create healthcare professionals that practice safe, equitable, competent care that safeguards patients and enables practitioners to deal with complexity and uncertainty [83].

However, there is evidence that medical curriculum enables a culture of discrimination and intolerance that adversely affects the lives of patients and healthcare professionals [84], especially those who identify as LGBTQ+ [33, 34]. Curriculum has also been described as a physical object that has its own power and through this power reinforces heteronorms in medical education [1, 85]. This conceptual understanding of medical curriculum as a perpetrator of violence is akin to epistemic-type violence towards oppressed peoples and as such, is not new. In fact, epistemic violence has been described in relation to oppression related to race and ethnicity [86, 87]. Some of the important ideas on which this argument is constructed include the concept that medical curriculum is historically acultural and that cultural identity is such a biased concept that it is not considered scientifically valid for diagnostic purposes [88].

Also, those who develop curriculum tend to be those in power and those who are less likely to have experience of intersectional oppression [89] and the type of experiences collected about patients predominates objective, quantitative data [90, 91]. Within this, voices from intersectionally oppressed individuals and those from the global south are excluded despite initiatives to increase their inclusion [90]. Moreover, interpretation of such data is biased to fit with historical, normative discourses about oppressed people [92]. These factors all feed into medical curriculum development and an ongoing, intersectional, epistemic violent cycle that forces individuals to live precariously.

The discrimination of LGBTQ+ healthcare professionals and students is also associated with institutional heteronormativity and hetero-discursive working environments. Heteronormativity is a normative discourse in medical schools [50, 93, 94] and is associated with LGBTQ+ healthcare students being ‘outed’, feeling that they are an invisible minority, and feeling excluded by the curriculum, medical leaders and peers [50, 93, 94]. This creates a hostile learning environment in which LGBTQ+ students are less likely to thrive [94]. A significant factor towards the perpetuation of this heteronormative discourse is the hidden curriculum [94]. Use of cis-sexist and heteronormative language and/or erasure of sexual and gender minoritised people outside of curriculum in medical schools devalues LGBTQ+ students lived experiences [94].

Wider literature on the subject suggests solutions to epistemic violence that could be applicable here. Razack et al. [95], suggest a reconsideration of curriculum based on shared humanity with peers, praxis and cultural safety. Dudar et al. [96], suggest an intersectional approach, using multiple teaching and assessment techniques. Finn et al. [97] suggest a model of curriculum based on advocacy and meaningful co-construction. We argue that the historic nature of oppression in medicine must be better articulated in curriculum with respect to issues such as HIV/AIDS. Furthermore, we argue that it should be considered how oppressed people globally feature in medical education and research [98] and that there is a need to re-consider how EDI-related institutional priorities are created and the potential for epistemic injustice in such decision-making processes [99].

### Minority Stress as a Daily Reality for LGBTQ+ Medical Students in the UK

Aligning with existing work [21, 100] we illustrated interconnected examples of minority stress (previously defined) in the participant population. Proximal (repercussions if LGBTQ+ status is known, closeting and internalised cis-heteronormativity), distal (observed and experienced discrimination) and general stressors (cis-heteronormative education environments, entanglements with differing cultural norms) were all demonstrated. These were connected to participants’ wellbeing in line with the theory and applied examples from the evidence base [101]. What this creates, in line with the theory of minority stress [21, 22], is an environment in which healthcare professionals and students are unable to be authentic and therefore comfortable to engage with healthcare services appropriately [76, 102]. This has an impact on how healthcare professionals care for patients, particularly those patients also experiencing intersectional oppression [103].

Although critiqued for its handling of intersectionality [104] the established nature of minority stress in LGBTQ+ inequality research provides a language to consider change. For instance, student-led organisations, curriculum developments, and multilevel (structural, relational and individual) interventions have been suggested to reduce minority stress in LGBTQ+ student populations internationally [105]. We argue that to enact change, multi-level interventions need to be adopted in tandem with intersectional coping resources (role models and allies) [105], and we argue a shift in medical school culture is needed internationally that recognises intersectional complexity and individuality.

### Strengths and Limitations

A limitation of this study was that we only drew on experiences from UK medical students. However, due to the paucity of qualitative data about this population, this approach was deemed appropriate. Furthermore, the large sample size and the considerable number of participants with experience of international medical education settings added to the generalisability of findings. Also, we drew on a considerable amount of evidence from multiple, international educational contexts to further add to the global transferability of the findings.

In addition to this, the methods carried limitations. The unstructured nature of the methods provided the opportunity for participants to contribute in any way they felt necessary. This left open the possibility of negativity bias which could have skewed the results. Aware of this limitation the researchers, who are experienced interviewers, supported participants to concentrate on balance, and reflection on change, rather than ruminating on negative experiences. We used ‘cue words’, picking up on words or phrases that could bring participants back to a more balanced understanding of their experiences. Examples of cue words were ‘change’ or ‘future’.

Furthermore, despite methodological mitigations, the lead researcher was known to participants to be a medical education professional, and this could have created a power imbalance. However, the combined epistemological approaches considered participant’s experiences from multiple perspectives and the concurrent reflexive process that was undertaken by the research team allowed us an enhanced awareness of biases and the impact of power imbalances on analysis.

## Conclusions and Suggestions for Future Research

In one of the biggest studies of its kind, the researchers enhanced understanding of the experiences of LGBTQ+ medical students using first person narratives as well as intersecting epistemologies and methodologies in a novel way. We made clear the necessity for a rigorous medical curriculum that balances scientific knowledge with person-centred learning and ongoing opportunities for professional identity formation internationally. We also demonstrated the complex lives of this population and how Materialism and Poststructuralism enable a better understanding of associated intersectional oppression and difficulties.

We argue that future research should consider the impact of Material entities, such as medical school buildings and dress codes on the experiences of minoritised groups internationally. Furthermore, we argue that research, curriculum and policy should put intersectional complexity at its core and consider how this shift could enable medical educators to support this student population to reach their potential as future physicians.

## Recommendations for Change

1. Medical education research needs to meaningfully engage with stakeholders’ voices to understand different perspectives on stakeholder experience. This could include undertaking and acting on equality impact assessments for all medical education research projects.
2. Multi-level educational interventions need to be adopted in tandem with intersectional coping resources to challenge epistemic violence and minority stress and better support students in medical schools internationally. This could include mentoring schemes and curriculum development based on shared humanity and humility.
3. Educators, policy makers and funders need to be more transparent about past and present oppressions and overtly include this throughout medical curriculums globally.
4. A shift in medical school culture is needed internationally to one that recognises intersectional complexity in a way that enables dynamic change in curriculum, healthcare policy, and practice for the benefit of all healthcare stakeholders. This could include meaningful co-production of curriculum change with students and patients.

## Supplementary Information

Below is the link to the electronic supplementary material.

Supplementary Material 1
