# Supplementary material for: Understanding the Experiences of LGBTQ+ Identifying Medical Students in the UK: A Multi-Institutional Qualitative Study
Source: Med Sci Educ. 2026 Apr 13;36(3):1605–20. doi: 10.1007/s40670-026-02715-6 (PMC13356126; doi:10.1007/s40670-026-02715-6)
Supplement: Supplementary file 1 — Supplementary Material 1 [file 40670_2026_2715_MOESM1_ESM.docx]

**Interview/ Narrative Prompts**

**1:1 Interview**

**Introduction:** Thank you for taking part in this project exploring the lived experiences of LGBTQ+ medical students in the UK. Today we are asking you to discuss with the researcher, Dr Bintley, what it’s like to be you; the good, the bad, and the ugly!

This is your space to discuss whatever you would like to discuss within the realms of the study aims and objectives. We would like to know about YOU, what medical school is and has been like so far, what it means to you to be a medical student and if and how your LGBTQ+ identity influences that (or doesn’t).

Just a note to reiterate that all responses are confidential within limits of health and safety. Your responses would only ever be shared beyond yourself and the researcher if it was felt that there was a threat to your health and wellbeing or that of others. I would also like to record this session if you are happy for me to do so. I also want to check that there isn’t anything else that you need in order to make the interview accessible or appropriate for you? Please let me know if there is anything.

I will be led by you how you would like this session to run. We can either just have a chat and see where it goes, I can ask you questions, you can ask yourself questions, or we can even talk around the subject if you would prefer.

If you are happy to continue? If it’s helpful I have a question that I offer to all participants as an opening question but we don’t have to use it.

If accepted: Tell me about you, who are you, for example?

**Prompts:**

- Can I invite you to tell me more about that in relation to your identity?
- Can I invite you to tell me more about the implications of that for your life outside of medical school?
- Can I ask if and how that relates to your experiences of placements and professional settings?
- What does it mean to be a medical student/ doctor/ professional do you think?
- Can I invite you to explore with me how and in what way are you figuring out the answers to the above question?
- Can I invite you to tell me about your experiences of being LGBTQ+ around your peers at medical school?
- Can I invite you to tell me about your experiences of being LGBTQ+ around your tutors, university lecturers and clinical teachers?
- Do you have any comments about the curriculum in which you are learning medicine?

**Template headings for the in-session summary of content:**

- Lived Experience of being an LGBTQ+ medical student in the UK
- Professional Identity Formation in relation to being an LGBTQ+ medical student in the UK
- Medical curriculum and Lived Experience of being an LGBTQ+ medical student in the UK
- The future for medical education in relation to LGBTQ+ medical students in the UK
- Any other comments

**Textual Narrative:**

**Introduction:** Thank you for taking part in this project exploring the lived experiences of LGBTQ+ medical students in the UK. Today we are asking you to discuss with the researcher, Dr Bintley, what it’s like to be you; the good, the bad, and the ugly!

This is your space to discuss whatever you would like to discuss within the realms of the study aims and objectives. We would like to know about YOU, what medical school is and has been like so far, what it means to you to be a medical student and if and how your LGBTQ+ identity influences that (or doesn’t).

Just a note to reiterate that all responses are confidential within limits of health and safety. Your responses would only ever be shared beyond yourself and the researcher if it was felt that there was a threat to your health and wellbeing or that of others. I also want to check that there isn’t anything else that you need in order to make the interview accessible or appropriate for you? Please let me know if there is anything.

I will be led by you how you would like the text to evolve. You could write prose or a poem, words in a pattern, a ‘brain dump’ of whatever text comes into your head or something completely different within the limits of the research question. Use as many, as little or no headings as you see fit, write what you want in whatever way you like. The only stipulation is that you write some words and these words explore the aims and objectives summarised above.

If you are happy to continue? If it’s helpful I have a question that I offer to all participants as an opening question but you don’t have to use it.

Tell me about you, who are you, for example?

If you get stuck you might find the following prompts useful:

- Might you consider in what way does what you are writing relate to who you are?
- Would you like to comment on what your experiences are of being a medical student who also identifies as LGBTQ+?
- Might you consider what the implications are of being a medical student on your life? Or the other way round?
- Can I invite you to think about whether there is any relation between what you are writing and your experiences of placements and professional settings?
- Might you want to consider what your writing tells you about yourself as a medical professional/ future medical professional?
- Can I invite you to tell me about your experiences of being LGBTQ+ around your peers at medical school?
- Can I invite you to tell me about your experiences of being LGBTQ+ around your tutors, university lecturers and clinical teachers?
- Do you have any comments about the curriculum in which you are learning medicine?
